# Redefining Beauty: Knowledge, Attitudes, and Behaviours Toward Aesthetic Medicine and Cosmetic Surgery in Urban Adults

**DOI:** 10.3390/clinpract16030047

**Published:** 2026-02-26

**Authors:** Fabiana Di Duca, Giancarlo Biondi, Elvira De Rosa, Alessandro Venuta, Salvatore Di Sarno, Alfonso Nardo, Bartolomeo Ferrante, Giovanni Mazzei, Stefano Scippa, Immacolata Russo, Maria Triassi, Paolo Montuori

**Affiliations:** 1 Department of Public Health, University “Federico II”, Via Sergio Pansini 5, 80131 Naples, Italy; fabianadiduca91@gmail.com (F.D.D.); alessandro.venuta@unina.it (A.V.);; 2 Department of Human Sciences and Quality of Life Promotion, San Raffaele University, 00166 Rome, Italy; 3 Department of Public Health, University Hospital of Naples “Federico II”, Via Sergio Pansini 5, 80131 Naples, Italy

**Keywords:** aesthetic medicine, plastic surgery, knowledge, attitude, behaviour, cross-sectional survey

## Abstract

Background: In recent years, aesthetic medicine and cosmetic surgery have seen significant growth, reflecting changing sociocultural views on beauty and self-care; however, public knowledge and awareness of associated risks remain inconsistent. This study aimed to assess knowledge, attitudes, and behaviours toward aesthetic medicine and cosmetic surgery in a large metropolitan population in Southern Italy using the Knowledge–Attitude–Practice (KAP) framework. Methods: A cross-sectional survey was conducted between June 2021 and January 2022 among 1079 adults aged 18–72 years residing in the metropolitan area of Naples. A structured questionnaire collected socio-demographic data and assessed knowledge, attitudes, and behaviours related to surgical and non-surgical aesthetic procedures. Descriptive statistics and multiple linear regression analyses were performed to identify predictors of knowledge, attitudes, and behaviours. Results: Overall, 66.8% of participants reported having undergone general beauty treatments, while 9.8% declared the use of cosmetic medicine procedures. A total of 5.1% had undergone botulinum toxin treatments, 11% reported filler injections, and 9.8% had experienced plastic surgery. A majority had strong knowledge, especially on non-surgical procedures, but there were gaps in their knowledge on side effects, regulations, age limits, and qualifications. Most viewed appearance as important, though with critical views of excessive aesthetic treatments and claimed limited social media influence. Female sex and parental status were positively associated with aesthetic behaviours, while attitudes emerged as the strongest predictor of engagement. Conclusions: Aesthetic practices are widely accepted within this urban population, yet important informational deficiencies persist. Targeted educational interventions based on the KAP framework are warranted to enhance health literacy, promote safe decision-making, and foster realistic expectations regarding aesthetic medicine and cosmetic surgery.

## 1. Introduction

### 1.1. Sociocultural Relevance and Psychological Dimensions

In contemporary societies, physical appearance has progressively gained relevance, becoming an important determinant of personal well-being, social interaction, and perceived self-worth [1,2]. The way individuals present themselves often represents the first element perceived in interpersonal relationships and may influence both social acceptance and professional opportunities [3]. Increasing attention to body image and appearance-related concerns has been associated with psychological dimensions such as self-esteem, confidence, and quality of life, particularly among young adults [4,5]. Dissatisfaction with one’s physical appearance has been linked to emotional distress, social withdrawal, anxiety, and depressive symptoms, highlighting the complex interplay between body image, mental health, and aesthetic practices [4,6,7]. In this context, aesthetic medicine and cosmetic surgery are increasingly perceived not merely as corrective interventions but as tools integrated into broader concepts of self-care and well-being [8].

### 1.2. Evolution of Aesthetic Medicine, Media Exposure, and Emerging Knowledge Gaps

Over recent decades, aesthetic medicine and cosmetic surgery have undergone a substantial transformation [9]. Advances in medical techniques have improved safety profiles and reduced recovery times, contributing to a shift from invasive surgical procedures toward minimally invasive treatments such as botulinum toxin injections and dermal fillers [10,11,12]. Epidemiological data indicate a steady global increase in the demand for aesthetic treatments, particularly minimally invasive procedures, which now far exceed surgical interventions in terms of volume [10]. Notably, the demographic profile of individuals seeking aesthetic treatments has evolved, with a progressive reduction in the average age of users and a predominance of female patients, although male participation is also increasing [13,14].

At the same time, exposure to aesthetic ideals through mass media and digital platforms has increased markedly [15,16]. While several studies suggest that social media may influence perceptions of beauty and motivate the pursuit of cosmetic procedures, the extent of this influence appears heterogeneous across populations and cultural contexts [17,18]. Despite the growing diffusion of aesthetic medicine, several studies have documented persistent gaps in public knowledge regarding the nature of cosmetic procedures, their potential risks, legal regulations, and the qualifications required to perform them [17,19]. Aesthetic medicine is a branch of modern medical practice that focuses on improving a person’s cosmetic appearance through non-surgical or minimally invasive procedures aimed at enhancing features such as skin quality, wrinkles, pigmentation, or volume loss [20]. Cosmetic surgery refers to surgical procedures that are performed to alter or enhance the appearance of bodily structures. Such surgeries are also elective and aesthetically motivated, but they are invasive and associated with longer recovery periods compared to aesthetic medicine procedures [21]. Misconceptions about side effects, professional roles, and eligibility criteria such as age limits or the need for psychological assessment have been reported in different populations [17,19,22]. These uncertainties may expose individuals to unsafe practices and unrealistic expectations [23].

In recent years, the aesthetic medicine market in Italy and across Europe has demonstrated robust and sustained growth, underpinned by both demand-side and supply-side dynamics. Market analyses indicate that the European aesthetic medicine sector is expanding significantly, with valuations forecasted to increase markedly over the coming decade and compound annual growth rates in the high single digits to low double digits, particularly in minimally invasive and non-surgical procedures [24,25] (Zhanna, 2025; MarketDataForecast, 2025). Italy stands out within the European context both for its high per capita rate of aesthetic treatments and for a notable increase in demand over the past decade, with certain reports indicating an increase in procedures of around 25% since the late 2000s [26] (Businesscoot, 2025).

Overall, these trends reflect a maturing and increasingly mainstream market in which aesthetic medicine is integrated into broader healthcare and wellness practices, supported by evolving consumer preferences, technological innovation, and regulatory frameworks that facilitate the adoption of new treatments [27] (Ramirez and Scherz, 2025).

### 1.3. Literature Gap, KAP Framework, and Study Aim

Existing research has largely focused on specific subgroups, such as university students or women seeking aesthetic procedures, or has examined isolated treatments like Botox or fillers [28,29,30]. Consequently, comprehensive investigations simultaneously assessing knowledge, attitudes, and behaviours across a broad and heterogeneous adult population remain limited. In particular, few studies have applied structured theoretical models capable of exploring how cognitive awareness interacts with psychological positioning and actual behavioural engagement.

In this context, the present study aims to investigate knowledge, attitudes, and behaviours related to aesthetic medicine and plastic surgery in a large metropolitan population in Southern Italy. By applying the Knowledge–Attitude–Practice (KAP) model to both surgical and non-surgical aesthetic treatments, this research seeks to identify demographic and psychosocial determinants associated with aesthetic practices and to provide evidence useful for the development of targeted educational and public health interventions.

## 2. Materials and Methods

### 2.1. Participants and Procedure

A cross-sectional study was carried out, from the beginning of June 2021 until the end of January 2022, through the administration of questionnaires, provided personally to citizens of the entire metropolitan city of Naples, Southern Italy (one of the largest and most densely populated cities in Italy). The distribution took place in schools as well as in widely accessible public venues, such as community centres and local cultural associations, in order to maximise participation and ensure representativeness. A non-probabilistic convenience sampling strategy was adopted, aiming to recruit participants from different social and educational backgrounds within the metropolitan area. Although efforts were made to ensure heterogeneity in age, education, and occupation, the sampling approach does not allow full representativeness of the general Italian population. The study was conducted in accordance with the principles of the Declaration of Helsinki. All participants were fully informed about the assurance of anonymity, the purpose of the research, and the intended use of the collected data. Participation was voluntary, with the guarantee that all information would be processed anonymously and confidentially, and that participants could withdraw at any time without providing a reason. Verbal informed consent was obtained prior to each interview. Each participant received a questionnaire directly (available upon request from the corresponding author), and at the time of completion, the study objectives as well as the procedures ensuring anonymity and privacy were explained both in written form through an introductory section of the questionnaire and verbally. Ethical approval was not required for this type of study, in accordance with local and national regulations (art. 12, paragraph 10, letter c of Legislative Decree No. 158 of 13 September 2012, converted with amendments into Law No. 189 of 8 November 2012, and Ministerial Decree of 8 February 2013).

The inclusion criteria encompassed both male and female participants, aged between 18 and 72 years, who were residents of the metropolitan area of Naples. The questionnaire collected basic demographic information from participants, including age, gender, education level, profession, smoking habits, and parental status (with specification of the number of children, if applicable). Smoking status was included as a general lifestyle indicator potentially associated with risk-related behaviours and health decision-making patterns. In addition, it comprised three distinct sections, focusing on knowledge, attitudes, and behaviours related to the appeal of aesthetic medicine and surgery, for a total of 36 questions (available upon request from the corresponding author). The inclusion or exclusion of specific elements within the knowledge, attitude, and behaviour domains was guided by the recommendations of the KAP Model [31]. The knowledge section included items assessing general awareness of side effects; however, it did not investigate procedure-specific short- and long-term complications in detail, as the focus was on overall risk perception rather than technical clinical knowledge. The development of the questionnaire took place by following a series of steps, listed here: (1) creation of the survey protocol; (2) preparation of the report; (3) monitoring of the evolution of the KAP survey in the field and (4) analysis of the data and drafting of the survey report. We reworked the questions to make them simpler, more understandable, and, above all, closed. Knowledge and Attitudes were assessed using a three-point Likert scale with response options of ‘Agree,’ ‘Uncertain,’ and ‘Disagree.’ In contrast, items concerning Behaviours employed a four-point format with choices of ‘Never,’ ‘Sometimes,’ ‘Often,’ and ‘Always.’ Each question was scored by assigning values from 1 to 3 for Knowledge and Attitudes, and from 1 to 4 for Behaviours. Correct responses were awarded the maximum score (3 for Knowledge and Attitudes, 4 for Behaviours), while incorrect responses received a score of 1. A pilot study was conducted to evaluate the structure and reliability of the questionnaire. All completed questionnaires were subsequently digitised, with coded responses entered into an Excel worksheet (MS Office) for analysis.

### 2.2. Statistical Analysis

The data included in the study were processed using the statistical software programme Statgraphics Centurion 18 (vers. 18.1.12). The analysis was conducted in two phases. In the first phase, descriptive statistics were applied, while in the second phase multiple linear regression analysis (MLRA) was performed. This technique allows the examination of relationships between dependent and independent variables, providing results of statistical significance (*p*-value < 0.05), the statistical significance of beta coefficients (*p*-value < 0.05), and the coefficient of determination (R^2^ and adjusted R^2^), which indicates the proportion of variance in the dependent variables explained by the independent variables.

Three MLRA models were developed: Knowledge regarding the consumption of beauty treatments (Model 1); Attitudes toward the practice of beauty treatments (Model 2); Actual Behaviour related to the habit of undergoing beauty treatments (Model 3). Dependent variables (Knowledge, Attitudes, and Behaviours) were obtained by summing the scores from the corresponding items, with inverse responses recoded accordingly. Independent variables included in all models were sex (1 = male, 2 = female), age (in years), marital status (1 = single, 2 = not single), level of education (1 = primary school, 2 = middle school, 3 = high school, 4 = graduation), occupation (1 = Lawyer, 2 = Doctor, 3 = Teacher, 4 = Student, 5 = Unemployed, 6 = Architect, 7 = Accountant, 8 = Law enforcement officer, 9 = Employee, 10 = Engineer, 11 = Entrepreneur, 12 = Trader, 13 = Worker, 14 = Other [specify]), and smoking status (1 = smoker, 2 = non-smoker/former smoker). In Model 2, Knowledge was added as an independent variable, while in Model 3 both Knowledge and Attitudes were included as independent variables. Attitudes and Knowledge were treated as indexes rather than scales, meaning that each observed variable (A1–A12 and K1–K12) was assumed to contribute to the associated latent constructs (Attitude and Knowledge). In this formative relationship, correlations among observed variables were not required. Conversely, the relationship between observed variables (B1–B15) and the latent construct Behaviour was considered reflective, with internal consistency confirmed by Cronbach’s alpha (α = 0.825). All statistical tests were two-tailed, and results were considered statistically significant at *p* ≤ 0.05.

## 3. Results

A total of 1133 questionnaires were distributed during the study period, of which 1079 were returned, yielding a response rate of 95.5%. This high level of participation underscores the robustness of the sample and its ability to represent the characteristics of a metropolitan population, not only within the Italian context but also in line with broader European urban demographics. The socio-demographic composition of the cohort reflects the diversity typical of large metropolitan areas (Table 1).

As shown in Table 1, women constituted the majority of respondents (73.3%), while men accounted for 26.7%. The mean age of participants was 30.4 years, with the youngest respondent aged 18 and the oldest 72. The age distribution was skewed toward younger adults, with 55.5% of the sample between 18 and 30 years, followed by 16.4% aged 31–35, 11.5% aged 36–40, 13.9% aged 41–60, and only 2.7% aged 61–72. Regarding smoking, 70.15% said they did not smoke, and regarding marital situation, 71.6% of the sample were single, and 28,4% were in a relationship. The majority (72.5%) had no children and those who had children had two at most (12.2%). Educational attainment was notably high among respondents. Nearly half of the sample (48.3%) held a high school diploma, while 47% had completed a university degree. Only a small minority reported primary (0.6%) or middle school education (4.1%). Occupational categories further illustrate the heterogeneity of the metropolitan setting. Students represented the largest group (31%), followed by physicians (17.8%) and teachers (10.2%). Other professions included employees (8.3%), lawyers (1.7%), engineers (0.9%), architects (0.6%), law enforcement officers (2%), traders (1.1%), workers (2.6%), and unemployed individuals (3.3%). A substantial proportion (18.4%) reported occupations classified as “other,” reflecting the broad spectrum of professional activities in a metropolitan environment. The respondents’ knowledge about beauty treatments is presented in Table 2.

In detail, the analysis of knowledge items revealed a generally high level of awareness among respondents, consistent with the elevated educational profile of the cohort. Most participants demonstrated familiarity with the basic definitions of common aesthetic procedures. For instance, 82.4% correctly identified filler as a hyaluronic acid injection used to increase volume, and 73.3% recognised botulinum toxin as the substance employed in Botox treatments. Similarly, 81% acknowledged that plastic surgeons primarily perform surgical procedures such as rhinoplasty and mammoplasty, while 80.6% correctly attributed cosmetic and make-up treatments to beauticians. Nevertheless, several areas of uncertainty emerged. More than one third of respondents (35.8%) expressed doubts about the potential side effects of botulinum toxin, and 57.1% incorrectly believed that its use has no adverse consequences. Confusion was also evident regarding the professional boundaries between beauticians and aesthetic physicians: 49.3% agreed that beauticians could perform filler injections but not Botox, while 33.2% remained uncertain. Legal and psychological aspects of cosmetic procedures also generated mixed responses. Only 39.8% agreed that plastic surgery is forbidden to minors, while 35.2% disagreed and 25% were uncertain, highlighting a lack of clarity regarding age restrictions. Similarly, opinions diverged on the necessity of psychological consultation prior to aesthetic interventions: 32.6% agreed it was mandatory for certain medical procedures, whereas 43.2% disagreed. In contrast, a majority (68.3%) recognised the requirement of psychological evaluation before undergoing plastic surgery. Table 3 describes attitudes toward beauty treatments.

The analysis of attitudes revealed a complex and sometimes contradictory set of perceptions among respondents. On the one hand, aesthetic concerns were recognised as highly relevant in daily life. A significant majority (91%) agreed that cosmetic defects can create discomfort, and 60.4% considered physical appearance an important facilitator of social integration and professional opportunities. Similarly, 39.5% acknowledged the importance of appearance in partner selection, underscoring the role of aesthetics in both personal and social domains. At the same time, respondents expressed a critical stance toward excessive reliance on aesthetic medicine. Only 9.9% agreed that people should undergo multiple beauty treatments, while 65.2% disagreed. Likewise, 64.2% rejected the idea that aesthetic medicine always improves appearance, and 79.1% disagreed with the notion that ageing inevitably requires aesthetic interventions. Interestingly, respondents strongly distanced themselves from the influence of social media: 93.6% disagreed with the statement that one must follow the beauty ideals proposed by these platforms. Other responses revealed ambivalence in the relationship between aesthetic medicine and lifestyle practices. Approximately 39.2% agreed that resorting to aesthetic medicine is easier than practicing sports, and 41.9% agreed that it is easier than following a healthy diet. Finally, more than half of the sample (53%) considered the costs of beauty treatments to be appropriate, indicating a general acceptance of the economic dimension of these practices. Table 4 shows data on behaviour towards beauty treatments.

Overall, 66.8% of respondents reported having undergone some form of beauty treatment, confirming the relevance of these practices in everyday life. However, only 9.8% indicated that they had undergone plastic surgery, while 5.1% reported Botox treatments and 11% filler injections. These figures reflect the predominance of non-surgical procedures. This can be explained by the fact that 55.5% of the respondents are very young, between 18 and 30 years old. Recommendation patterns varied: 27.3% reported having recommended beauty treatments, 3.9% often, 24.1% sometimes, while 44.7% never recommended them. In contrast, plastic surgery recommendations were less frequent, with 16% reporting yes, 1.2% often, 17.4% sometimes, and 65.3% never. Specific procedures were less common. Botox treatments were reported by 3.8% of respondents, 0.6% often, 0.7% sometimes, and 94.8% never. Filler treatments were reported by 7.8% regularly, 1.2% often, 2% sometimes, and 89% never. Regarding expenditure, 11% of respondents reported spending more than 150 euros per month on personal care, 6.9% often, 23.7% sometimes, and 58.4% never. Judging the physical appearance of others was reported by 8.2% regularly, 7.3% often, 54% sometimes, and 30.5% never. Lifestyle indicators were also assessed. Practicing sports was reported by 26.9% regularly, 12.3% often, 43.9% sometimes, and 16.9% never. Following a healthy diet was reported by 27.2% regularly, 30% often, 35.2% sometimes, and 7.5% never. Finally, 20.2% of respondents stated that they need at least one hour in the morning to get ready, 11.3% often, 27.7% sometimes, and 40.8% never. Table 5 illustrates the results of multiple logistic regression analysis (MLRA) in three models.

Model 1 showed that knowledge about beauty treatments was significantly and positively associated with age, number of children, and level of education (*p* < 0.005), while no significant correlations were observed with sex, marital status, or smoking status. Model 2 indicated that attitudes toward beauty treatments were negatively correlated with being single and with having children (*p* < 0.005), whereas a positive correlation was found with the overall knowledge index (*p* < 0.005), confirming that higher knowledge scores were linked to more favourable attitudes. Model 3 demonstrated that behaviours related to beauty treatments were negatively correlated with being single (*p* < 0.001), and positively correlated with sex, number of children, and attitudes (*p* < 0.05), with no significant association with smoking status.

## 4. Discussion

The objective of this study is to identify a target population to whom an educational programme aimed at improving knowledge, promoting safe decision-making, and fostering realistic expectations about aesthetic medicine and surgery could be addressed. The application of the Knowledge–Attitude–Practice (KAP) framework represents a methodological strength of this study. Rather than limiting the analysis to descriptive awareness of procedures, the KAP model allows for the examination of the internal coherence between cognitive knowledge, psychological positioning, and behavioural engagement. This approach makes it possible to detect discrepancies between what individuals believe they know and how they actually act, offering insights that purely prevalence-based or media-exposure studies cannot provide. In this regard, our findings offer several insights into how demographic characteristics, knowledge levels, attitudes, and behaviours converge to shape the population’s relationship with cosmetic procedures.

As expected, given the high educational level of our sample, understanding of aesthetic medicine treatments was generally strong, and the overall knowledge of participants was generally good. Specifically, 82.4% of respondents knew that fillers consist of hyaluronic acid injections aimed at increasing volume, while 73.3% correctly identified Botox as an injection of botulinum toxin. These results are consistent with findings reported in other studies available in the literature [18,28]. Participants largely agreed that general awareness of aesthetic medicine is higher among women, a perception that aligns with findings reported in the previous literature, including the study of Bondagji et al. [32]. The multiple linear regression analysis further confirmed a statistically significant positive association between educational attainment and knowledge (*p*-value = 0.02). This finding contrasts with other studies in the literature reporting weaker or nonsignificant correlations [18,22]; however, it is consistent with evidence from other investigations, such as those conducted by Fareed et al. [30]. An additional statistically significant relationship was found between knowledge and participants with children. Another discrepancy concerns the understanding of Botox: in our study, 73.3% correctly recognised Botox as an injection of botulinum toxin, whereas in other research such as the study by Alenezi et al. the reference population showed greater uncertainty on this topic [22]. Despite the overall good level of knowledge regarding aesthetic medicine treatments and their applications, substantial doubts persist concerning their potential side effects [17,33]. As shown in Table 2, 57.1% of participants believed that these procedures have no side effects, while only 7.1% disagreed with this statement. Uncertainties surrounding the safety of aesthetic treatments such as Botox and fillers have also been reported in other studies, including that of Alenezi et al. [22]. In his investigation of knowledge and attitudes among the Saudi population, where the use of Botox has markedly increased, only a small proportion of respondents recognised botulinum toxin as a potentially hazardous biological substance [22]. Similarly, Alradaddi et al., in a study examining the increasing demand for cosmetic treatments, highlighted that risk perception remains highly heterogeneous within the target population [17]. Further confusion emerges regarding the identification of professionals qualified to perform aesthetic procedures. As shown in Table 2, 49.3% of participants agreed with the statement that beauticians are allowed to perform filler injections but not Botox, while 33.2% reported uncertainty on this topic. Similar findings were reported in the study by Alradaddi et al., where a substantial proportion of respondents failed to clearly identify the professional figures authorised to administer such treatments [17]. Likewise, Zargaran’s analysis of practitioners performing aesthetic procedures in the United Kingdom revealed that a large proportion of providers are not medically trained, contributing significantly to public confusion regarding who is appropriately qualified to carry out these interventions [19]. Doubts also remain as to whether or not one has to be of legal age to be able to perform plastic surgery and whether or not psychological counselling is necessary. It is important to clarify that, according to Italian regulations, injectable aesthetic procedures such as botulinum toxin and dermal fillers are classified as medical acts and must be performed by licenced physicians. The questionnaire included items referring to beauticians not to suggest legal permissibility, but to assess public awareness and misconceptions regarding professional qualifications. These insights are relevant for identifying knowledge gaps and informing educational interventions. Since professional regulations vary internationally, the findings regarding perceptions of non-medical providers may not be directly generalizable to other healthcare systems. The assessment of attitudes highlights how physical appearance plays a fundamental role in the daily lives of participants, influencing factors such as partner choice and personal self-esteem. The importance of physical appearance in romantic selection has also been explored by other authors, including Aladwan et al., who, in a study involving individuals willing to undergo aesthetic medicine treatments, identified the main motivations for seeking such procedures as appearing more attractive, enhancing self-esteem, and correcting perceived aesthetic defects [1]. The desire to correct physical flaws may therefore represent a key determinant in the decision to resort to aesthetic treatments; in fact, approximately 91% of our sample believed that aesthetic defects could generate a sense of personal discomfort. Similarly, Hong et al. reported that dissatisfaction with one’s body may lead to depression and anxiety, regardless of sex [4]. However, aesthetic medicine is not perceived as the sole or inevitable solution for improving physical appearance. Indeed, 64.2% of respondents disagreed with the statement that aesthetic medicine always improves appearance, and 65.2% did not believe that individuals should undergo an increasing number of aesthetic medicine treatments. Furthermore, 93.6% of participants did not perceive social media influence as a determining factor in the decision to undergo aesthetic medicine treatments. This finding is in stark contrast with studies such as that by Alradaddi et al., who reported a strong influence of social media on individuals’ decisions to pursue cosmetic procedures [17]. Conversely, our results are consistent with those of Aladwan et al. and Kadhim et al., whose studies similarly suggest that social media does not play a major role in shaping attitudes toward aesthetic medicine [1,18]. However, although exposure to aesthetic content through digital platforms has increased dramatically in recent years, information availability does not automatically translate into structured health literacy. The widespread familiarity observed in our sample with procedures such as fillers and botulinum toxin likely reflect media exposure and procedural visibility. However, the persistence of misunderstandings regarding potential side effects, legal age restrictions, and professional qualifications indicates that procedural awareness may coexist with incomplete risk perception and regulatory knowledge. This discrepancy highlights the difference between passive information consumption and critical understanding, suggesting that digital dissemination alone is insufficient to ensure informed decision-making. Physical appearance also appears to play a role in facilitating integration within social and occupational contexts, as approximately 60.4% of respondents agreed with this statement. Similar findings were reported by Sarpila et al., who examined the association between physical appearance and perceived occupational congruence [3]. In that study, more attractive individuals were judged as more “fit” for their jobs, with effects varying according to gender and professional field. Moreover, the authors highlighted how such perceptions may contribute to the reinforcement or widening of inequalities within the labour market [3]. Responses to questions comparing aesthetic medicine with physical activity and a balanced diet revealed a rather heterogeneous pattern, highlighting how all these factors are perceived as important components in maintaining psychological and social well-being. This finding is consistent with previous research, such as the study by Miko et al., which emphasises the central role of lifestyle factors in overall health and well-being [8]. Finally, we explored participants’ perceptions of the costs associated with aesthetic medicine. More than half of respondents (53%) considered the costs of aesthetic treatments to be appropriate, a result that contrasts with the findings of Alradaddi et al., who identified cost as one of the main barriers to the uptake of aesthetic procedures [17]. While economic accessibility is often cited as a determinant influencing the choice of providers, including non-medical professionals, our findings suggest that cost perception alone does not fully explain aesthetic behaviours within this population. The coexistence of relatively positive cost perception and persistent knowledge gaps indicates that economic convenience and informational limitations may operate simultaneously rather than as alternative explanations. Future research should further explore how financial factors interact with perceived competence, trust, and risk awareness in shaping decision-making processes. The results of Model 2 indicate that attitudes toward aesthetic treatments are mainly influenced by relational and family-related factors. Being single and having children were both significantly and negatively associated with a favourable attitude. This finding contrasts with other studies in the literature, in which being married has been associated with more positive attitudes toward aesthetic medicine [13,34]. Conversely, a higher level of knowledge was positively associated with a more favourable attitude, confirming the central role of information in shaping opinions toward aesthetic medicine. Demographic variables such as sex, age, educational level, and smoking habits did not show significant effects, indicating that attitudes are more strongly driven by psychosocial dimensions rather than by structural characteristics.

The analysis of behaviours shows that participants resort far more frequently to general beauty treatments than to aesthetic medicine procedures or plastic surgery. This pattern can be largely explained by the age distribution of our sample, as approximately 55% of respondents were between 18 and 30 years old. At this stage of life, individuals tend to focus more on non-invasive beauty practices aimed at personal care and well-being rather than on medical or surgical aesthetic interventions. This finding is consistent with data from authoritative sources such as the American Society of Plastic Surgeons (ASPS), which consistently report that the majority of aesthetic medicine and cosmetic surgical procedures are performed in individuals over 30 years of age, with the highest prevalence observed in the 35–50 and older age groups [10]. According to these databases, minimally invasive procedures such as botulinum toxin injections and hyaluronic acid fillers are predominantly utilised after the third decade of life, often as part of anti-ageing strategies. Therefore, the younger age of our study population likely accounts for the lower uptake of aesthetic medicine and plastic surgery observed, while explaining the greater reliance on beauty treatments and lifestyle-related practices. The importance of aesthetic appearance is further evidenced by the fact that more than 50% of participants reported having recommended beauty treatments to others, whereas over 60% have never advised others to undergo cosmetic surgery. These findings align with global procedural trends showing a clear preference for minimally invasive aesthetic procedures over surgical ones. According to the ASPS statistics, minimally invasive treatments such as neuromodulator injections (e.g., Botox) and hyaluronic acid fillers accounted for tens of millions of procedures worldwide, far outnumbering surgical interventions [10]. In 2024 alone, nearly 28.5 million minimally invasive procedures were performed, including nearly 9.9 million neuromodulator injections and over 5.3 million filler treatments, a volume more than double that of cosmetic surgical procedures [10]. Further evidence of the importance attributed to personal appearance emerges from daily self-care habits. More than 50% of participants reported spending over one hour each morning preparing themselves, and a substantial proportion of these individuals also declared spending more than 150 euro per month on personal care. These behaviours highlight a significant investment of both time and financial resources in appearance-related practices. Moreover, the frequent engagement in physical activity and adherence to a balanced diet, as reported by a large share of respondents, reinforce the broader concept of personal well-being. The results of Model 3 highlight a set of significant associations that allow the identification of the profile of individuals most inclined to engage in aesthetic practices. First, behaviour was positively associated with female sex, confirming findings from the international literature indicating that women represent the predominant consumers of aesthetic treatments, both minimally invasive and surgical [32,35]. This result is also consistent with the composition of our sample, in which women accounted for more than 70% of participants. Another relevant finding is the positive correlation between attitudes and behaviour, suggesting that greater psychological openness toward aesthetic medicine translates into a higher likelihood of actually undergoing treatments. This association has also been reported by other authors, including Alduosari et al. and Adedeji et al., who demonstrated that favourable attitudes are a significant predictor of cosmetic treatment uptake [15,36]. Furthermore, aesthetic behaviours were more frequent among participants with children. This association may be explained by body changes related to pregnancy and childbirth and by the desire to restore pre-pregnancy physical appearance [37,38]. The finding that having children predicts engagement in aesthetic practices may be partly explained by the bodily changes associated with pregnancy and childbirth. Postpartum alterations, including changes in body shape, weight distribution, skin elasticity, and musculoskeletal tone, can influence self-perception and body image [39,40] (Owusu et al., 2025; Marano et al., 2025). In addition, psychological and social pressures on mothers to restore their pre-pregnancy appearance, including societal expectations and cultural ideals of postnatal body image, may further motivate engagement in aesthetic or self-care practices [41] (Rodgers et al., 2024). Several studies have shown that these changes may motivate individuals to adopt aesthetic or self-care practices to restore or enhance perceived physical appearance, support psychological well-being, or regain a sense of personal identity after childbirth [42,43] (Whitaker et al., 2025; Mohammad et al., 2023). Incorporating this perspective provides a more comprehensive understanding of the relationship between parenthood and engagement in aesthetic behaviours. Conversely, being single was negatively associated with aesthetic behaviour, suggesting that relational context may influence motivation to invest in aesthetic treatments [44,45]. From a broader public health perspective, aesthetic medicine should not be considered merely a consumer-driven or lifestyle phenomenon. It represents a health-related practice involving medical risk, regulatory oversight, psychological vulnerability, and ethical considerations. In this context, identifying discrepancies between perceived knowledge and actual understanding becomes essential to prevent unsafe practices, unrealistic expectations, and potential long-term psychological consequences. Structured educational interventions, rather than reliance on unregulated digital information, may therefore play a key role in promoting safe and informed engagement with aesthetic treatments.

## 5. Limitations

This study presents some limitations that should be acknowledged when interpreting the findings. First, the cross-sectional design does not allow causal inferences to be drawn between knowledge, attitudes, and behaviours toward aesthetic medicine and cosmetic surgery. Associations identified through regression analyses describe correlations but cannot establish temporal or causal relationships. Second, data were collected through self-reported questionnaires, which may be subject to recall bias and social desirability bias, particularly for sensitive topics such as aesthetic practices, body image, and lifestyle behaviours. Furthermore, as the study was conducted in a single metropolitan area in Southern Italy, cultural and regional differences may limit the generalizability of the findings to other contexts. Attitudes toward aesthetic medicine may vary according to local socioeconomic conditions, healthcare regulations, and cultural norms, and multicentre or cross-national studies would be valuable to further explore these differences. The sample was skewed toward younger adults and women, which may have influenced the observed prevalence of behaviours and attitudes. Additionally, the use of a convenience sampling strategy may limit the representativeness of the findings. Although the sample was large and socio-demographically diverse, it was not selected through a probabilistic or stratified design. Therefore, caution is warranted when generalising the results beyond the studied metropolitan context. Finally, while the questionnaire was developed according to the KAP model and showed good internal consistency for the behaviour domain, knowledge and attitude were treated as formative indexes rather than validated psychometric scales, which may limit comparability with other studies using different instruments. Furthermore, knowledge regarding complications was explored only at a general level and did not include detailed procedure-specific adverse events. Future studies should incorporate more granular assessments of complication awareness to better evaluate risk literacy.

## 6. Policies

Despite these limitations, the findings of this study have relevant implications for public health policies and preventive strategies. The persistence of knowledge gaps regarding side effects, legal regulations, age limits, and professional qualifications highlights the need for structured educational interventions aimed at improving health literacy in the field of aesthetic medicine. Public health authorities should promote clear, accessible, and evidence-based information campaigns to help individuals make informed and safe decisions, particularly in urban populations and among younger age groups increasingly exposed to aesthetic ideals. Policies should also reinforce regulatory frameworks and communication strategies that clearly define the roles and qualifications of professionals authorised to perform aesthetic procedures, thereby reducing the risk of unsafe practices. Moreover, integrating education on body image, realistic expectations, and the psychological dimensions of aesthetic treatments into broader health promotion programmes could help prevent excessive medicalisation of beauty and mitigate potential mental health consequences. From a policy perspective, aesthetic medicine should be addressed not only as a consumer-driven phenomenon but also as a public health issue requiring coordinated actions involving healthcare providers, institutions, and educational systems.

## 7. Conclusions

The study examines knowledge, attitudes, and behaviours toward aesthetic medicine and plastic surgery in a large urban population, overcoming previous research limitations focused on specific subgroups or single procedures. Aesthetic treatments, particularly minimally invasive ones, are widely used—especially among women and younger individuals—and increasingly seen as part of everyday well-being. Overall knowledge is high, particularly among those with higher education or children, but gaps remain regarding side effects, regulations, and professional qualifications, highlighting the need for targeted educational interventions. Attitudes reflect a critical perspective: appearance is important socially and professionally, yet there is resistance to over-medicalisation and social media pressures. Greater knowledge correlates with more balanced attitudes. Behaviourally, non-invasive treatments are preferred, especially among younger participants; women and parents engage more frequently, while single individuals show less favourable attitudes and behaviours. Attitudes are the strongest predictor of behaviour, more than demographics. The study’s strength lies in its large, heterogeneous sample and the application of the KAP model to both surgical and non-surgical practices. Future research should explore the long-term psychological, social, and health impacts of aesthetic engagement, and public health strategies should target young adults, parents, and those with lower education to promote safe, informed decisions and a conception of beauty aligned with overall well-being.

## Figures and Tables

**Table 1:** Study population characteristics.

Study Population	N	Percentage (%)
Sex		
Male	288	26.7
Female	791	73.3
Age		
18–30	599	55.5
31–35	177	16.4
36–40	124	11.5
41–60	150	13.9
61–72	29	2.7
Education		
Primary School	7	0.6
Middle school	44	4.1
High school	521	48.3
Degree	507	47.0
Profession		
Lawyer	18	1.7
Architect	6	0.6
Engineer	10	0.9
Physician	192	17.8
Business consultant	8	0.7
Business owner	15	1.4
Teacher	110	10.2
Law enforcement	22	2.0
Dealer	12	1.1
Student	334	31
Employee	90	8.3
Worker	28	2.6
Unemployed	36	3.3
Other	198	18.4
Single		
Yes	306	28.4
No	773	71.6
Children		
Yes	297	27.5
No	782	72.5
Number of Children		
0	9	0.8
1	102	9.5
2	132	12.2
3	58	5.4
4	2	0.2
5	2	0.2
Smoker		
Yes	318	29.5
No	761	70.5

**Table 2:** Respondents’ knowledge of beauty treatments.

N.	Statement (Variables)	Agree (%)	Uncertain (%)	Disagree (%)
K1	In 70% of cases, women undergo cosmetic medicine treatment.	66.1	25.2	8.7
K2	Filler consists of hyaluronic acid injection to increase volume.	82.4	12.0	5.6
K3	Lip filler is the most widespread cosmetic medicine treatment.	62.8	29.6	7.6
K4	Botox is an injection of botulinum toxin.	73.3	23.3	3.4
K5	The use of botulinum toxin has no side effect.	57.1	35.8	7.1
K6	Beauty treatments such as cosmetics and make-up are carried out by the beautician.	80.6	9.3	10.1
K7	Non-aesthetic treatments are carried out by the aesthetic doctor such as filler and Botox.	75.4	15.9	8.6
K8	The plastic surgeon performs mainly surgical aesthetic treatments such as rhinoplasty and mammoplasty.	81.0	9.5	9.5
K9	The beautician can perform fillers but not Botox.	49.3	33.2	17.5
K10	Plastic surgery is forbidden to minors.	39.8	25.0	35.2
K11	A psychological consultation is manatory to carry out interventions with aesthetic medicine.	32.6	24.2	43.2
K12	A psychological consultation is mandatory to carry out plastic surgery.	68.3	19.3	12.4

**Table 3:** Attitude of respondents toward beauty treatments.

N.	Statement (Variables)	Agree (%)	Uncertain (%)	Disagree (%)
A1	People should undergo multiple beauty treatments.	9.9	24.8	65.2
A2	The natural look is preferable to that obtained with the aesthetic medicine.	17.8	29.0	53.2
A3	Aesthetic medicine always improves the appearance.	9.7	26.0	64.2
A4	Physical appearance is important when choosing a partner.	39.5	28.5	32.1
A5	Cosmetic defects can create discomfort.	91.0	8.9	0.1
A6	Accepting your physical defects is a safety indicator.	10.0	19.1	70.9
A7	You must always follow the idea of beauty proposed by social media.	3.1	3.3	93.6
A8	As we get older, it is inevitable to make aesthetic treatments.	6.2	14.7	79.1
A9	A beautiful physical appearance facilitates social integration and work opportunities.	60.4	24.6	15.0
A10	It is easier to resort to aesthetic medicine than to practice sports.	39.2	20.9	39.9
A11	It is easier to resort to cosmetic medicine than to follow a healthy diet.	41.9	18.5	39.6
A12	The costs of beauty treatments are right.	53.0	46.9	0.1

**Table 4:** Behaviour of respondents toward beauty treatments.

N.	Questions	Yes (%)	Often (%)	Sometimes (%)	Never (%)
B1	Do you resort to beauty treatments?	30.1	8.4	28.3	33.2
B2	Do you use cosmetic medicine?	7.2	2.0	7.9	82.9
B3	Do you use plastic surgery?	5.3	0.6	3.9	90.2
B4	Have you ever recommended beauty treatments?	27.3	3.9	24.1	44.7
B5	Have you ever recommended plastic surgery treatments?	16.0	1.2	17.4	65.3
B6	Do you get Botox treatments?	3.8	0.6	0.7	94.8
B7	Do you get filler treatments?	7.8	1.2	2.0	89.0
B8	Do you spend more than 150 euros per month for the care of your person?	11.0	6.9	23.7	58.4
B9	Do you judge the physical appearance of others?	8.2	7.3	54	30.5
B10	Do you practice sports?	26.9	12.3	43.9	16.9
B11	Do you follow a healthy diet?	27.2	30.0	35.2	7.5
B12	In the morning, you need at least an hour to get ready.	20.2	11.3	27.7	40.8

**Table 5:** Results of the linear multiple regression.

	Non-Standardised Coefficients		Standardised Coefficients		
	B	Standard Error	Beta	t	*p*-Value
Model 1—Dependent variable: Knowledge					
Sex	0.223	0.515	0.025	0.432	0.666
Age	0.037	0.019	0.132	1.949	0.052
Single	0.635	0.734	−0.51	−0.866	0.387
Children	2.770	0.983	0.179	2.820	0.005
How many children	0.274	0.171	0.099	1.603	0.110
Education	0.600	0.250	0.140	2.400	0.020
Smoke	−0.448	0.380	−0.069	−1.180	0.239
Model 2—Dependent variable: Attitudes					
Sex	0.998	0.571	0.890	1.750	0.081
Age	0.001	0.021	0.030	0.440	0.965
Single	−5.550	0.813	−0.357	−6.829	0.000
Children	−3.767	1.103	−0.195	−3.420	0.001
How many children	−0.834	0.190	−0.240	−4.280	0.000
Education	−0.178	0.280	−0.340	−0.630	0.526
Smoke	0.020	0.421	−0.003	0.048	0.962
K tot	0.283	0.064	0.227	4.390	0.000
Model 3—Dependent variable: Behaviour					
Sex	3.252	1.000	0.149	3.250	0.001
Age	0.044	0.037	0.065	1.196	0.233
Children	5.970	1.961	0.160	3.044	0.003
Single	−9.510	1.530	−0.316	−6.233	0.000
How many children	−0.329	0.342	−0.049	−0.961	0.338
Education	1.196	0.489	0.118	2.445	0.150
Smoke	−1.025	0.735	−0.066	−1.390	0.164
K tot	0.217	0.116	0.090	1.870	0.610
A tot	0.636	0.100	0.329	6.278	0.000

## Data Availability

The original contributions presented in this study are included in the article. Further inquiries can be directed to the corresponding author.
